# Dimerization of DOCK2 Is Essential for DOCK2-Mediated Rac Activation and Lymphocyte Migration

**DOI:** 10.1371/journal.pone.0046277

**Published:** 2012-09-26

**Authors:** Masao Terasawa, Takehito Uruno, Sayako Mori, Mutsuko Kukimoto-Niino, Akihiko Nishikimi, Fumiyuki Sanematsu, Yoshihiko Tanaka, Shigeyuki Yokoyama, Yoshinori Fukui

**Affiliations:** 1 Division of Immunogenetics, Department of Immunobiology and Neuroscience, Medical Institute of Bioregulation, Kyushu University, Fukuoka, Japan; 2 Research Center for Advanced Immunology, Kyushu University, Fukuoka, Japan; 3 Core Research for Evolutional Science and Technology (CREST), Japan Science and Technology Agency, Kawaguchi, Japan; 4 RIKEN Systems and Structural Biology Center, Yokohama, Japan; 5 Department of Biophysics and Biochemistry, Graduate School of Science, The University of Tokyo, Tokyo, Japan; Hungarian Academy of Sciences, Hungary

## Abstract

The migratory properties of lymphocytes depend on DOCK2, an atypical Rac activator predominantly expressed in hematopoietic cells. Although DOCK2 does not contain the Dbl homology domain typically found in guanine nucleotide exchange factors (GEFs), DOCK2 mediates the GTP-GDP exchange reaction for Rac via its DOCK homology region (DHR)-2 (also known as CZH2 or Docker) domain. DOCK2 DHR-2 domain is composed of three lobes, and Rac binding site and catalytic center are generated entirely from lobes B and C. On the other hand, lobe A has been implicated in dimer formation, yet its physiological significance remains unknown. Here, we report that lobe A-mediated DOCK2 dimerization is crucial for Rac activation and lymphocyte migration. We found that unlike wild-type DOCK2, DOCK2 mutant lacking lobe A failed to restore motility and polarity when expressed in thymoma cells and primary T cells lacking endogenous expression of DOCK2. Similar results were obtained with the DOCK2 point mutant having a defect in dimerization. Deletion of lobe A from the DHR-2 domain did not affect Rac GEF activity in vitro. However, fluorescence resonance energy transfer analyses revealed that lobe A is required for DOCK2 to activate Rac effectively during cell migration. Our results thus indicate that DOCK2 dimerization is functionally important under the physiological condition where only limited amounts of DOCK2 and Rac are localized to the plasma membrane.

## Introduction

Cell migration is a fundamental biological response involving membrane polarization and cytoskeletal dynamics [1]. In response to chemoattractants, filamentous actin (F-actin) polymerizes asymmetrically at the leading edge of the cell, providing the force necessary to extend membrane protrusion in the direction of migration [2]. This morphologic polarity is regulated by Rac, a member of the small GTPases that cycle GDP-bound inactive and GTP-bound active states [3]. Like other small GTPases, conversion of the GDP-bound Rac to the active state is catalyzed by guanine nucleotide exchange factors (GEFs). The classical GEFs share the Dbl-homology (DH) domain to mediate the GTP-GDP exchange reaction [3]. Until recently, DH-domain containing proteins have been considered to be the universal GEFs in eukaryotes. However, accumulating evidence indicates that the evolutionarily conserved DOCK family proteins act as major GEFs in varied biological settings. In mammals, DOCK family proteins consist of 11 members, all of which contain a conserved DOCK homology region (DHR)-2 (also know as CZH2 or Docker) domain and mediate the GTP-GDP exchange reaction for Rho family of small GTPases through this domain [4], [5], [6].

Lymphocytes are highly motile leukocytes, and play central roles in acquired immune responses to invading pathogens. Lymphocytes differentiate in primary lymphoid organs, and migrate into secondary lymphoid tissues such as the lymph nodes and spleen via the blood. This migration process of lymphocytes is regulated by chemokines such as CCL21, CCL19, CXCL13, and CXCL12, and these chemokines are expressed on stromal cells and vascular or lymphatic endothelial cells [7]. When chemokines bind to their receptors, heterotrimeric G protein is dissociated into α- and βγ-subunits, which activates a variety of signaling pathways including Rac. In lymphocytes, chemokine-induced Rac activation is critically dependent on DOCK2, an atypical Rac activator predominantly expressed in hematopoietic cells [8], [9]. As a result, DOCK2-deficient (DOCK2^−/−^) lymphocytes exhibit a severe defect in chemotactic responses [9], [10], [11], [12]. Although DOCK2 does not contain the DH domain, DOCK2 catalyzes the GTP-GDP exchange reaction for Rac by means of its DHR-2 domain [4], [5]. DOCK2 DHR-2 domain is composed of three lobes (lobes A, B, and C), and Rac binding site and catalytic center are generated entirely from lobes B and C [13]. On the other hand, lobe A has been implicated in dimer formation of DOCK2 and DOCK9 [13], [14], [15], yet its physiological significance remains unknown. In this study, we examined whether lobe A-mediated DOCK2 dimerization is functionally important for Rac activation and lymphocyte migration.

## Results and Discussion

**Figure 1:**
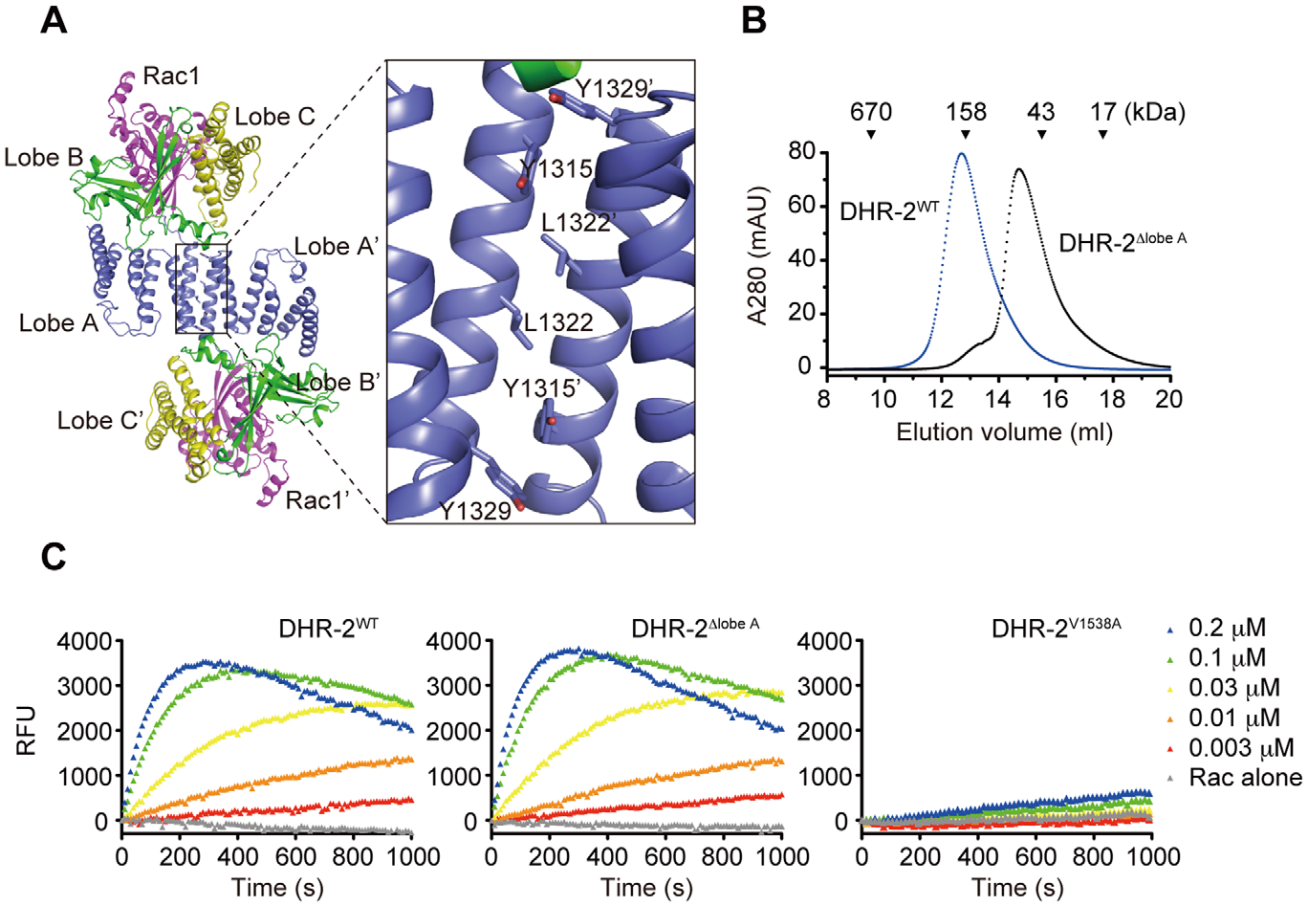
The lobe A is required for dimerization, but not for the Rac GEF activity of DHR-2 domain. (A) Ribbon diagram of the human DOCK2^DHR-2^-Rac1 complex. Overall structure (left) and dimerization interface (right) are shown. The coordinates and structure factors have been deposited in the Protein Data Bank (www.pdb.org) under accession code 3B13. (B) Size-exclusion chromatography analysis for DHR-2^WT^ and DHR-2^Δlobe A^. For protein size estimation, their elution volumes were compared with those of protein standards, thyroglobulin (670 kDa), immunoglobulin G (158 kDa), ovalbumin (43 kDa), and myoglobulin (17 kDa). (C) The Rac GEF activity was compared among DHR-2^WT^, DHR-2^Δlobe A^, and DHR-2^V1538A^ using BODIPY-FL-GTP.

A recent structure of human DOCK2 DHR-2 complexed with Rac indicates that, while Rac binding site and catalytic center are generated entirely from lobes B and C, lobe A mediates dimerization of DHR-2 domain [13]. This was also confirmed in our structural analysis (Figure 1A) [16]. Consistently, size-exclusion chromatography revealed that murine DOCK2 DHR-2 mutant lacking lobe A (DHR-2^Δlobe A^) fails to dimerize (Figure 1B). The valine residue at position 1538 of DOCK2 DHR-2 is known to function as a nucleotide sensor [13]. When this residue was mutated to alanine (designated DHR-2^V1538A^), the Rac GEF activity was almost completely lost (Figure 1C). However, deletion of lobe A from DOCK2 DHR-2 did not affect the Rac GEF activity in vitro (Figure 1C), as recently reported [17].

**Figure 2:**
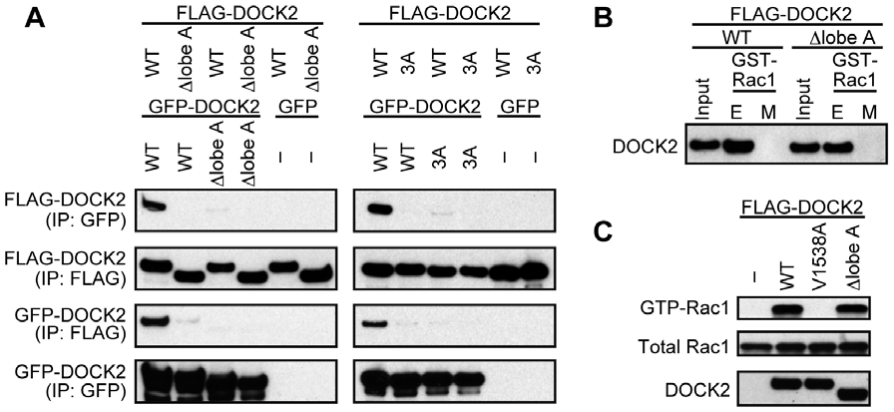
DOCK2 forms homodimer via lobe A of DHR-2 domain in cells. (A) Following expression of FLAG-tagged WT or mutant DOCK2 (Δlobe A or 3A) in HEK-293T cells in combination with GFP-tagged WT or mutant DOCK2 (Δlobe A or 3A) or GFP alone, cell extracts were immunoprecipitated with anti-FLAG M2 antibody or anti-GFP antibody. Immunoblotting was carried out to detect homodimerization using the relevant antibodies. (B) Extracts from HEK-293T cells expressing FLAG-tagged WT DOCK2 or Δlobe A were pulled down with GST-fusion Rac1. Assays were done in Tris-buffered saline-Tween-20 supplemented with 10 mM EDTA (E) or 10 mM MgCl_2_ plus 30 µM GTPγS (M). (C) Following expression of FLAG-tagged WT DOCK2 or Δlobe A in HEK-293T cells, Rac activation was analyzed. In (A-C), data are representative of, at least, three independent experiments.

To further examine whether full-length DOCK2 dimerizes via lobe A, we simultaneously expressed FLAG- and green fluorescent protein (GFP)-tagged wild-type (WT) or mutant DOCK2 in HEK-293T cells. Reciprocal immunoprecipitation experiments using the antibody specific for each tag revealed that DOCK2 forms homodimer (Figure 2A). However, such homodimerization was not induced when DOCK2 mutant lacking lobe A (Δlobe A) was expressed as either form (Figure 2A). Contacts at the human lobe A interface involve aromatic and aliphatic residues such as tyrosine-1315, leucine-1322, and tyrosine-1329 (Figure 1A). When the corresponding amino acid residues of murine DOCK2 were mutated to alanine, this 3A mutant failed to form homodimer (Figure 2A). These results indicate that lobe A is also important for dimerization of DOCK2 in cells.

Having found that full-length DOCK2 dimerizes via lobe A in cells, we next examined whether the Δlobe A mutant retains the ability to bind to Rac. For this purpose, extracts of HEK-293T cells expressing FLAG-tagged WT DOCK2 or Δlobe A were pulled down with glutathione-S transferase (GST)-fusion Rac1 in the presence of 10 mM EDTA or 10 mM MgCl_2_ plus 30 µM GTPγS. These experiments revealed that Δlobe A as well as WT DOCK2 binds selectively and efficiently to the nucleotide-free Rac1 (Figure 2B). Consistent with this finding, Rac activation was comparably induced by overexpression of WT DOCK2 and Δlobe A in HEK-293T cells (Fig. 2C). Thus, Δlobe A retains the ability to bind to Rac and to mediate the GTP-GDP exchange reaction for Rac when overexpressed in HEK-293T cells.

**Figure 3:**
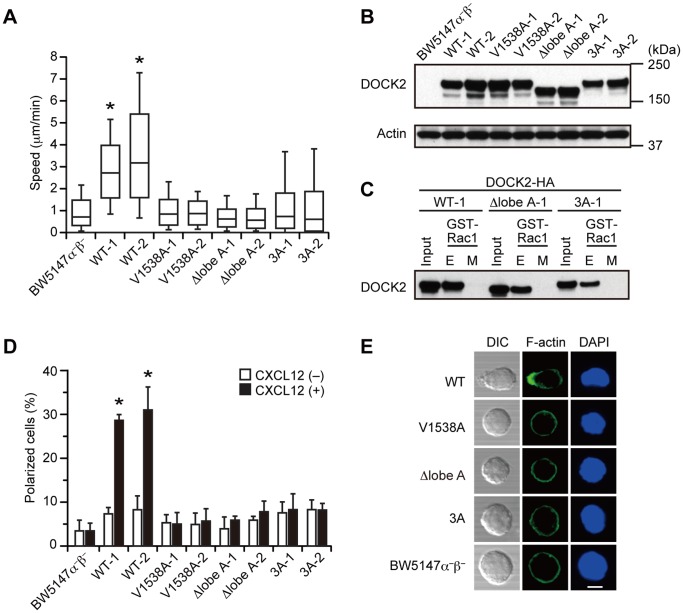
The expression of the Δlobe A and 3A mutant fails to restore motility and polarity in BW5147α^–^β^–^ cells. (A) Migration assays for BW5147α^–^β^–^ cells stably expressing HA-tagged WT or mutant DOCK2 (V1538A, Δlobe A, or 3A). At least 145 cells were analyzed for each category of cells. Each box plot exhibits the median (central line within each box), the 25th and 75th percentile values (box ends) and the 10th and 90th percentile values (error bars). Statistical analysis was performed by the Kruskal-Wallis H test followed by the Mann-Whitney U test with Bonferroni correction. *P<0.01 for comparison with BW5147α^–^β^–^ cells. Data are representative of, at least, two independent experiments. (B) The expression levels of HA-tagged WT or mutant DOCK2 (V1538A, Δlobe A, or 3A) in BW5147α^–^β^–^ cells. Actin expression was included as a loading control. (C) Extracts from BW5147α^–^β^–^ cells expressing HA-tagged WT or mutant DOCK2 (Δlobe A or 3A) were pulled down with GST-fusion Rac1. Assays were done in Tris-buffered saline-Tween-20 supplemented with 10 mM EDTA (E) or 10 mM MgCl_2_ plus 30 µM GTPγS (M). Data are representative of two independent experiments. (D, E) BW5147α^–^β^–^ cells stably expressing WT or mutant DOCK2 (V1538A, Δlobe A, or 3A) were stimulated in suspension with CXCL12, and stained with phalloidin and DAPI. Data indicate the percentage of polarized cells (mean ± SD of at least 160 cells) (D) with representative images (E). *P<0.01 by Student’s t-test for comparison with unstimulated cells. Scale bar, 5 µm.

To examine the role of DOCK2 dimerization under more physiological conditions, we stably expressed WT or mutant DOCK2 in BW5147α^–^β^–^ thymoma cells lacking the expression of DOCK2 [9], [16]. Although BW5147α^–^β^–^ cells were unable to migrate efficiently on stromal cells prepared from the lymph nodes, the expression of WT DOCK2, but not the V1538A mutant, significantly improved the motility of these cells (Figure 3A), indicating that DOCK2 regulates migratory response of BW5147α^–^β^–^ cells through Rac activation. We then analyzed the motility of BW5147α^–^β^–^ cells expressing Δlobe A or 3A. The expression level of these mutants was comparable to that of WT DOCK2 in two independent clones (Figure 3B). In addition, both Δlobe A and 3A showed definite binding to the nucleotide-free Rac1 in pull-down assays (Figure 3C). These results indicate that the Δlobe A and 3A mutants expressed in BW5147α^–^β^–^ cells can potentially act as Rac GEFs. Surprisingly, however, the expression of Δlobe A or 3A in BW5147α^–^β^–^ cells failed to restore the motility (Figure 3A). BW5147α^–^β^–^ cells express CXCR4, a chemokine receptor for CXCL12. When BW5147α^–^β^–^ cells expressing WT DOCK2 were stimulated in suspension with CXCL12, they exhibited polarized morphology with focused distribution of F-actin (Figure 3D,E). However, such morphologic polarity was again not induced in BW5147α^–^β^–^ cells expressing Δlobe A or 3A (Figure 3D,E).

**Figure 4:**
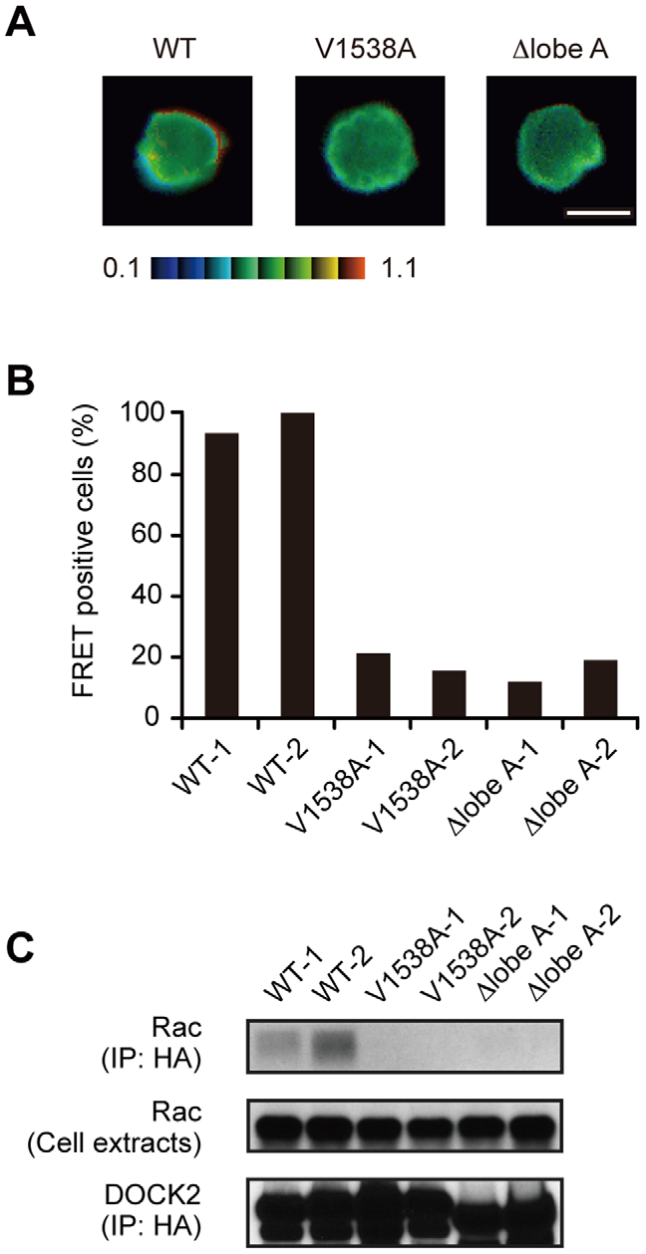
Lobe A-mediated DOCK2 dimerization is required to activate Rac effectively during cell migration. (A, B) FRET analyses for Rac activation in BW5147α^–^β^–^ cells stably expressing HA-tagged WT or mutant DOCK2 (V1538A or Δlobe A). Cells were retrovirally transduced with Raichu-Rac, and were loaded on stromal cells prepared from the lymph nodes. After 4 hours of incubation, images were taken every 30 seconds, and the emission ratio of 527 nm/475 nm (FRET/CFP ratio) was used to represent the FRET efficiency. The FRET/CFP ratios at the plasma membrane were normalized by dividing by the lowest value in the cells, and cells were judged FRET-positive when the ratio was above 1.6. Data indicate the percentage of FRET-positive cells (B) with representative images (A). For each category of cells, 12–19 cells were analyzed. Scale bar, 5 µm. (C) The association of DOCK2 with Rac in BW5147α^–^β^–^ cells stably expressing HA-tagged WT or mutant DOCK2 (V1538A or Δlobe A). Cell extracts were incubated with anti-HA affinity matrix in the presence of 10 mM EDTA, and bound Rac was detected with anti-Rac1 antibody.

Having found that the expression of Δlobe A and 3A was unable to restore motility and polarity in BW5147α^–^β^–^ cells, we next examined activation of Rac in BW5147α^–^β^–^ cells by retrovirally transducing Raichu-Rac [18], the fluorescence resonance energy transfer (FRET)-based biosensor to monitor Rac activation at the membrane. Comparison of the FRET efficiency revealed that Rac was activated at the leading edge membrane in the case of BW5147α^–^β^–^ cells expressing WT DOCK2 (Figure 4A,B). However, such Rac activation was scarcely found in BW5147α^–^β^–^ cells expressing Δlobe A as well as those expressing V1538A (Figure 4A,B). Consistent with this finding, the association between DOCK2 and endogenous Rac was detected in BW5147α^–^β^–^ cells expressing WT DOCK2, but not those expressing Δlobe A or V1538A (Figure 4C). These results indicate that lobe A-mediated DOCK2 dimerization is required to bind to and activate Rac effectively in BW5147α^–^β^–^ cells.

**Figure 5:**
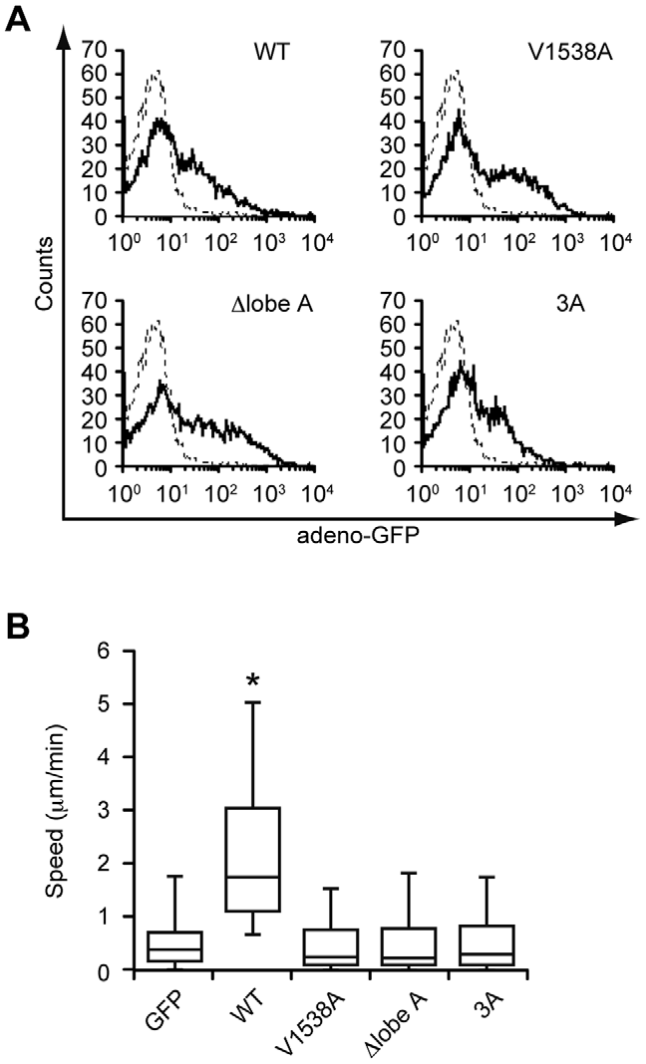
DOCK2 dimerization is functionally important for migration of primary T cells. (A) Flow-cytometric profiles for *Dock2^−/−^* T cells infected with recombinant adenovirus encoding GFP-tagged WT or mutant DOCK2 (V1538A, Δlobe A, or 3A). Dotted lines indicate the control profile of uninfected cells. (B) Migration assays for *Dock2^−/−^* T cells expressing GFP-tagged WT or mutant DOCK2 (V1538A, Δlobe A, or 3A). At least 140 GFP-positive cells were analyzed for each category of cells. Each box plot exhibits the median (central line within each box), the 25th and 75th percentile values (box ends) and the 10th and 90th percentile values (error bars). Statistical analysis was performed by the Kruskal-Wallis H test followed by the Mann-Whitney U test with Bonferroni correction. *P<0.01 for comparison with the control samples expressing GFP alone.

Finally, we wanted to test whether DOCK2 dimerization is also important in primary T cells. For this purpose, we crossed DOCK2-deficient (*Dock2^−/−^*) mice with transgenic mice expressing the gene encoding coxsackie-adenovirus receptor (CAR) under the Lck promoter [9], [19], and developed the experimental system with which full-length DOCK2 can be readily expressed in *Dock2^−/−^* T cells by adenoviral transfer (Figure 5A). When GFP-tagged WT DOCK2 was expressed in *Dock2^−/−^* T cells, the migration speed markedly increased, compared with that of the control expressing GFP alone (Figure 5B). However, similar to the case of V1538A, the expression of Δlobe A and 3A did not improve the motility of *Dock2^−/−^* T cells (Figure 5B).

In this study, we have shown that lobe A-mediated DOCK2 dimerization is important for Rac activation and lymphocyte migration. We found that lobe A is dispensable for the GTP-GDP exchange activity of the DHR-2 domain in vitro, and that DOCK2 mutants having a defect in dimerization retain the ability to bind to the nucleotide-free Rac in pull-down assays. However, the expression of these mutants in BW5147α^–^β^–^ cells and *Dock2^−/−^* T cells failed to restore Rac activation and migratory responses. Our results thus provide the first evidence for the physiological significance of DOCK2 dimerization in DOCK2-mediated cellular functions. The precise mechanism of how DOCK2 dimerization regulates the efficacy of Rac activation in cells is currently unknown. However, it seems likely that DOCK2 dimerization enhances signal transduction by recruiting Rac to the same signaling complex and by increasing local concentration of active Rac at plasma membrane, as was suggested for protein dimerization in other systems [20]. Interestingly, it has been reported that the DH domains of the classical GEFs also form oligomers, and the mutations causing a defect in oligomerization impaired their biological activities [21], [22], [23]. Therefore, oligomerization may be a common feature in GEFs to facilitate their signaling activities in cells where only limited amounts of target GTPases are available.

## Materials and Methods

### Ethics Statement

The protocol of animal experiments was approved by the committee of Ethics of Animal Experiments of Kyushu University (Permit Number: A24-016-0). All efforts were made to minimize suffering during the experiments.

### Mice

*Dock2^−/−^* mice and CAR-expressing transgenic mice have been described previously [9], [19]. Mice were kept under specific pathogen-free conditions in the animal facility of Kyushu University.

### Size-exclusion Chromatography

Proteins were chromatographed on a Superdex 200 10/300 GL column (GE Healthcare, Piscataway, NJ) connected to an AKTA FPLC system (GE Healthcare) using 20 mM Tris-HCl (pH 8.0) containing 250 mM NaCl and 5 mM 2-mercaptoethanol as running buffer at a flow rate of 1 ml/minute at 20°C.

### In vitro GEF Assays

The gene encoding DOCK2 DHR-2 domain or its mutants was cloned into the pET-SUMO vector to express fusion protein [17]. For measurement of the Rac GEF activity, GDP-loaded GST-Rac1 was incubated with BODIPY-FL-GTP (Invitrogen, Carlsbad, CA) for 5 minutes at 30°C. After equilibration, WT DOCK2 DHR2 (DHR-2^WT^) or its mutants (DHR-2^Δlobe A^ and DHR-2^V1538A^) were added to the mixtures, and the change of BODIPY-FL fluorescence (excitation  = 488 nm, emission  = 514 nm) was monitored at 30°C using a XS-N spectrofluorimeter (Molecular Devices, Sunnyvale, CA).

### Plasmids, Transfection, and Cell Culture

The genes encoding FLAG- or hemagglutinin (HA)-tagged DOCK2 and its mutants were created with pBJ1 vector [24]. The genes designed to express GFP- or FLAG-tagged DOCK2 and its mutants under the ubiquitin promoter were subcloned into pENTR3C (Invitrogen) before use. These plasmids were transfected by electroporation or with polyethylenimine to BW5147α^–^β^–^ thymoma cells [25] or HEK-293T cells (RIKEN BioResource Center; RCB2202), respectively. The adenoviral vector pAd-DOCK2 was generated by specific recombination of pENTR3C-DOCK2 with pAd/PL-DEST (Invitrogen). The pAd-DOCK2 with or without mutations was transfected into HEK-293A cells (Invitrogen) to amplify the recombinant adenovirus. The resultant virus was purified with cesium chloride centrifugation, and was used to infect CAR-expressing *Dock2^−/−^* T cells. Cells were then stimulated with immobilized anti-CD3 (20 µg/ml; eBioscience, San Diego, CA) and anti-CD28 (2 µg/ml; eBioscience) antibodies in RPMI 1640 medium supplemented with 10% fetal calf serum, IL-2 (100 U/ml; PeproTech, Rocky Hills, NJ), and IL-7 (20 ng/ml; PeproTech). After 24 hours of incubation, viable cells were recovered using Lympholyte-M (Cedarlane, Ontario, Canada) and suspended in Leibovitz L-15 medium (Invitrogen) for migration assays.

### Immunoprecipitation, Pull-down Assays, and Immunoblotting

Cell extracts were immunoprecipitated with anti-FLAG M2 antibody (Sigma-Aldrich, St Louis, MO), anti-GFP antibody (Invitrogen), or anti-HA antibody (Roche Applied Science, Basel, Switzerland). For pull-down assays, extracts of cells expressing FLAG- or HA-tagged WT or mutant DOCK2 were incubated with GST-Rac1 immobilized on glutathione beads (50% suspension) in binding buffer (20 mM Tris-HCl, 150 mM NaCl, 0.1% Tween-20, pH 7.5) supplemented with 10 mM EDTA (buffer E) or with 10 mM MgCl_2_ plus 30 µM GTPγS (buffer M) at 4°C for 60 minutes. To assess activation of Rac1, aliquots of the cell extracts were incubated with GST-fusion Rac/Cdc42-binding domain of PAK1 (Millipore) at 4°C for 60 minutes. Immunoblotting was carried out with anti-DYKDDDDK tag antibody (Wako, Osaka, Japan; for detection of FLAG-tagged proteins), anti-GFP antibody (Santa Cruz Biotechnology, Santa Cruz, CA), anti-HA antibody (Roche Applied Science), or anti-Rac antibody (Millipore, Billerica, MA).

### Migration Assays

Stromal cells were prepared from the peripheral lymph nodes as previously described [16] and stimulated with tumor necrosis factor-α (10 ng/mL; PeproTech) the day before assays. Primary T cells or BW5147α^–^β^–^ cells expressing WT or mutant DOCK2 were placed on a monolayer of stromal cells in the presence or absence of CCL21 (100 nM; R&D Systems, Minneapolis, MN). After 4 hours of incubation, phase-contrast images were obtained every 30 seconds for 20 minutes at 37°C on an IX-81 inverted microscope (Olympus, Tokyo, Japan). Migration of individual cells was tracked using the MetaMorph imaging software (Molecular Devices), and the migration speed was calculated by dividing the total path length by the total assay time.

### Immunofluorescence Microscopy

BW5147α^–^β^–^ cells stably expressing WT or mutant DOCK2 were stimulated with CXCL12 (400 ng/ml; R&D Systems) for 4 minutes and then fixed with 4% paraformaldehyde. Cells were treated with 0.1% Triton X-100 for 5 minutes, and incubated with Alexa Fluor 488-conjugated phalloidin (Invitrogen) and 4-diamino-2-phenylindole (DAPI; Wako) to stain F-actin and nuclei, respectively. Images were taken with a laser scanning confocal microscopy (LSM 510 META; Carl Zeiss, Heidelberg, Germany).

### FRET

The retroviral vector pMXs was used to create the plasmid encoding Raichu-Rac [18]. This plasmid was transfected into Platinum-E packaging cells [26] to generate the recombinant retrovirus. Retroviral infection was performed as previously described [27]. FRET (excitation 440 nm/emission peak 527 nm) and CFP (excitation 440 nm/emission peak 475 nm) images were obtained by using an Olympus IX-81 inverted microscope. After recordings were made, ratio images of FRET/CFP were created with MetaMorph and were used to represent the efficiency of the FRET.
